# The Microstructure, Antibacterial and Antitumor Activities of Chitosan Oligosaccharides and Derivatives

**DOI:** 10.3390/md20010069

**Published:** 2022-01-13

**Authors:** Dawei Yu, Jiayao Feng, Huimin You, Shipeng Zhou, Yan Bai, Jincan He, Hua Cao, Qishi Che, Jiao Guo, Zhengquan Su

**Affiliations:** 1 Guangdong Engineering Research Center of Natural Products and New Drugs, Guangdong Provincial University Engineering Technology Research Center of Natural Products and Drugs, Guangdong Pharmaceutical University, Guangzhou 510006, China; ydw65038@163.com (D.Y.); fjy54525452@163.com (J.F.); yhm98815@163.com (H.Y.); zszs64235@163.com (S.Z.); 2 Guangdong Metabolic Disease Research Center of Integrated Chinese and Western Medicine, Key Laboratory of Glucolipid Metabolic Disorder, Ministry of Education of China, Guangdong TCM Key Laboratory for Metabolic Diseases, Guangdong Pharmaceutical University, Guangzhou 510006, China; 3 School of Public Health, Guangdong Pharmaceutical University, Guangzhou 510310, China; angell_bai@163.com (Y.B.); hejincan300@163.com (J.H.); 4 School of Chemistry and Chemical Engineering, Guangdong Pharmaceutical University, Zhongshan 528458, China; caohua@gdpu.edu.cn; 5 Guangzhou Rainhome Pharm & Tech Co., Ltd., Science City, Guangzhou 510663, China; cheqishi@rhkj.com.cn

**Keywords:** chitosan oligosaccharide, derivatives, antimicrobial, antitumor

## Abstract

Chitosan obtained from abundant marine resources has been proven to have a variety of biological activities. However, due to its poor water solubility, chitosan application is limited, and the degradation products of chitosan oligosaccharides are better than chitosan regarding performance. Chitosan oligosaccharides have two kinds of active groups, amino and hydroxyl groups, which can form a variety of derivatives, and the properties of these derivatives can be further improved. In this review, the key structures of chitosan oligosaccharides and recent studies on chitosan oligosaccharide derivatives, including their synthesis methods, are described. Finally, the antimicrobial and antitumor applications of chitosan oligosaccharides and their derivatives are discussed.

## 1. Introduction

Marine resources have the potential to be used in the development of high-value products. Polysaccharide biomaterials represented by chitin and chitosan, collagen-based materials derived from marine biological composites, have been studied extensively [1]. Marine resources are far more abundant and available than is currently recognized, and in recent studies, it has been proposed that there may be marine polysaccharides that can treat COVID-19 [2]. There are a large number of crustacean arthropods, such as shrimp and crab, living in the ocean. The content of chitin extracted from their shells is second only to that of cellulose from land resources. Chitin is deproteinized and deacetylated in an alkaline environment to obtain chitosan. Chitosan is the only basic cationic polysaccharide in nature. To date, chitosan has been found to have a variety of biological activities, such as antibacterial [3,4,5], antitumor [6,7], antioxidant [5] and anti-Alzheimer’s disease [8,9] activities, and has been used as a drug carrier [10,11,12] or as a prebiotic to promote human health [13]. Chitosan can play a role in medicine and food, but it can also be used to remove pollutants form aqueous environments. Chitosan and its composites are attracting considerable interest as environmentally acceptable adsorbents and have the potential to remove many of these contaminants [14].

The long chain structure of chitosan leads to the occurrence of sugar chain entanglement, and the intermolecular and intramolecular hydrogen bonds are very strong [15]. As a result, the physical and chemical properties of chitosan are poor and the solubility in common solvents is low [16]. High viscosity and other problems limit the use of chitosan. To solve the disadvantages of chitosan, researchers have degraded chitosan to obtain chitosan oligosaccharides (COSs).

The concept of “shell biorefining” was proposed and developed for the extraction of nitrogen-containing chemicals with extensive use of crustacean shells. The proposed concept indicated that COSs, polymeric polysaccharides with abundant sources, have broad prospects for utilization [17].

To confirm the safety of COSs, a large number of studies have proven that COSs are nontoxic substances with high safety. In 2016, a rat study found that COSs did not damage liver mitochondria or lung tissue function [18,19]. In a study of the effect of COSs on colorectal cancer in a mouse model, there was no significant toxicity of COSs to the kidney and liver at oral doses [20]. In 2019, a study exhibited that COSs show no signs of toxicity to human sperm function [21].

The monomer of COSs is similar to D-glucose and replaces the hydroxyl group on C2 of D-glucose with an amino or acetamide group. The overall molecular structure is unequally linked by D-N-acetylglucosamine and D-2-glucosamine via β-1,4 glycosidic bonds. Compared with chitosan, the degree of acetylation (DA) of COSs is less than 10%, the degree of polymerization (DP) is less than 50–55, and the average molecular weight (MW) is less than 10 kDa. The relationship between the biological activity and the physical and chemical properties of COSs is directly related to the MW; the charge distribution of the DA, DP and N-glucosamine units; and the water solubility. COSs have been reported to have low viscosity and high intestinal epithelial absorption [22,23].

To date, a large number of structural modifications of COSs have been reported, and the structure of COSs can be artificially modified by means of chemical synthesis to further increase their biological activity. The modification sites of COSs concentrate on the two types of active groups, hydroxyl groups and amino groups. Generally, acetylation, alkylation, quaternization, or reaction with aldehydes and ketones occur on the amino group, while the hydroxyl group can undergo reactions such as acetylation, cross-linking, graft and so on. Xiaoli Liu et al. [24,25] synthesized a COS-N-chlorokojic acid mannich base (COS-N-MB) polymer based on COSs that had better antioxidant and antibacterial properties than free COSs and kojic acid. Lin Yue et al. [26] synthesized three kinds of COS-O-cinnamyl alcohol (Cin) compounds with different degrees of substitution (DSs) using Cin, and they exhibited higher thermal stability, better antimicrobial activity and lower crystallinity than COSs.

In this paper, the current research in terms of the derivatization methods of COSs, the structural changes after derivatization, especially the changes in amino groups after the introduction of groups, and the implementation of COSs and their derivatives in antibacterial and antitumor applications are reviewed, and the reasons for the enhanced antibacterial and antitumor activity after derivatization are explained.

## 2. Synthesis and Structural Attributes of COS

### 2.1. Key Structural Properties of COSs

**Figure 1:**
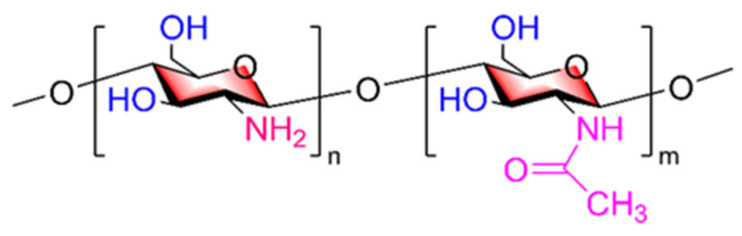
Key structural characteristics of COSs (n > m).

In the review of Anant S. Balijepalli and Mark W. Grinstaff [27], it was proposed that carbohydrates have their key structural characteristics. COSs, which are also carbohydrates, have diversified biological activities due to their unique chemical structure. The D-N-acetylglucosamine and D-2-glucosamine units in the main chain of COSs are functionalized by hydrophilic hydroxyl groups and amino groups and hydrophobic acetamide groups. The reason for the rigid pyran ring in COSs is that the whole structure is restricted by torsion, forming a tightly arranged linear structure. COS molecules have two types of active groups that serve as modification sites, the primary hydroxyl group on C6, the secondary hydroxyl group on C3, and the amino group on C2, exhibiting the properties of primary alcohols, secondary alcohols, and primary amines, respectively (Figure 1). In general, the amino group is acetylated, alkylated, or quaternized or reacts with aldehydes and ketones, while the hydroxyl group can undergo reactions such as acetylation, cross-linking, grafting, and other reactions, broadening its application range.

The oxygen atom in the hydroxyl group of the oligosaccharide and the nitrogen atom in the amino group are both unequally sp^3^-hybridized. The oxygen atom has two unshared electron pairs located in the two sp^3^ hybridized orbitals, and the remaining two sp^3^-hybridized orbitals form O-C and O-H σ bonds with C5 and hydrogen atoms, respectively. In the hydroxyl group, due to the difference in electronegativity between atoms, the electron clouds of both C-O and O-H bonds are biased toward the oxygen atoms, forming a polar covalent bond. The lone pair electrons on hydroxyl oxygen atoms can accept protons and have certain alkalinity and nucleophilicity. Nitrogen, compared to oxygen, has only one unshared pair occupying one of the sp^3^ orbitals, and the other three sp^3^ orbitals each have one electron. Similarly, unshared electron pairs, which have a tendency to share the pair of electrons with other atoms, are alkaline and nucleophilic, and can bind a proton through coordination bonds, leading to protonation.

A variety of biological activities of COSs are related to the MW, DP, DA, density and distribution of the cation charge; moreover, the amino group content, chelation and solubility of COSs are related to the biological activities of COSs, and amino groups can play a variety of roles in the form of noncovalent bonding with the corresponding targets in vivo.

Compared with oxygen atoms, nitrogen atoms have weaker electronegativity, and the amino group is more nucleophilic than the hydroxyl group. Although the nucleophilic properties of the two moieties are different, they can still react with electrophiles. COSs can be divided into two categories: those that undergo selective reactions and those that undergo nonselective reactions. COSs are formed by cleaving the glycosidic bond of chitosan. By comparing the infrared spectra of the two types of COSs, it can be seen that the chemical environment in which the key groups are located does not change. Therefore, the selective modification strategy of chitosan is helpful for the synthesis of chitosan derivatives [28,29]. For the synthesis of some special derivatives, COSs with MWs less than 10 kDa have not been directly used. For these derivatives, the strategy used in chitosan can be used analogously for reference.

### 2.2. Acylated COS Derivatives

**Figure 2:**
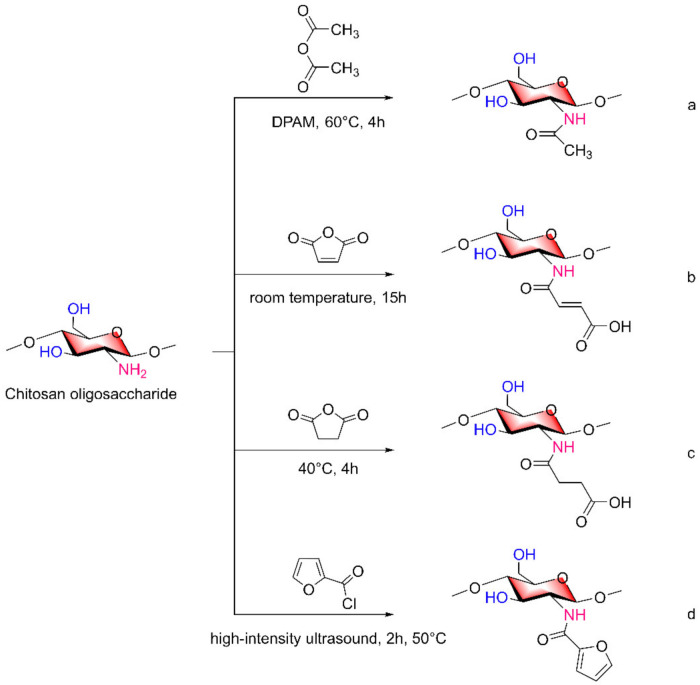
Synthesis of acylated COS derivatives. (**a**) 60 °C for 4 h to obtain highly acetylated COSs, (**b**) room temperature for 15 h to obtain N-maleoyl COS, (**c**) 40 °C for 4 h to obtain N-succinyl COS, (**d**) ultrasound, 50 °C for 2 h to obtain N-furanyl COSs.

Amino and hydroxyl groups on COSs are able to undergo acylation reactions. Based on their properties, these moieties can also be used to shield amino groups so that the amino groups are not affected in the reaction process, which will be described in detail in the following sections. The simplest derivatives of acylated COSs are N-acetyl COSs. Cui Hao and Lixia Gao et al. [30] reacted acetic anhydride with COSs to prepare partially acetylated COSs and fully acetylated COSs. Qingsong Xu et al. [31] also used acetic anhydride as an acylation reagent, 4-dimethylaminopyridine (DMAP) as a catalyst, and a COS stirring reaction at 60 °C for 4 h to obtain highly acetylated COSs (NaCOSs, Figure 2a). In addition to acetic anhydride, there are other reagents that can be used as acylation reagents, such as maleic anhydride and succinic anhydride. Tao Sun et al. [32] prepared N-maleoyl COS (Figure 2b) and N-succinyl COS (Figure 2c) with the same degree of substitution.

In addition to anhydride reactions, acylation can also occur with substances that have a carbonyl structure. Di Zhao et al. [33] obtained N-furanyl COSs (NF-COSs, Figure 2d) by acylation of COSs dissolved in acetic acid and furfuryl chloride under high intensity ultrasound at 50 °C for 2 h.

### 2.3. Alkylated COS Derivatives

According to reports in the literature, the reaction method used in the synthesis of alkylated COSs depends on the structure of the alkylation reagent. Common alkylating reagents are halohydrocarbons, alcohols, carbonyl compounds. When halohydrocarbons and alcohols are used as alkylating agents, they undergo nucleophilic substitution reaction with COSs. When ketones are used, COSs need to undergo nucleophilic addition reaction with them at first, and then lose water to form ketimines. Then the ketimine intermediates are reacted by strong reducing agents such as sodium hydrogen borate to generate alkylated COSs. The azomethine -N = CH- group in the Schiff base structure has good donor properties in coordination chemistry, and can form very stable complexes with a variety of transition metal ions [34]. At present, the synthesis and application of chitosan-Schiff bases (CSBs) have been reported in detail, and the synthesis strategy can be transferred to the synthesis of CSBs [35].

Sodium alginate was oxidized to alginate aldehyde and spontaneously cross-linked with the amino group of COSs to form a Schiff base, and the ZnO nanoparticles were loaded to form sodium alginate-COS-zinc oxide (SA-COS-ZnO) composite hydrogel, which was used to promote wound healing [36].

**Figure 3:**
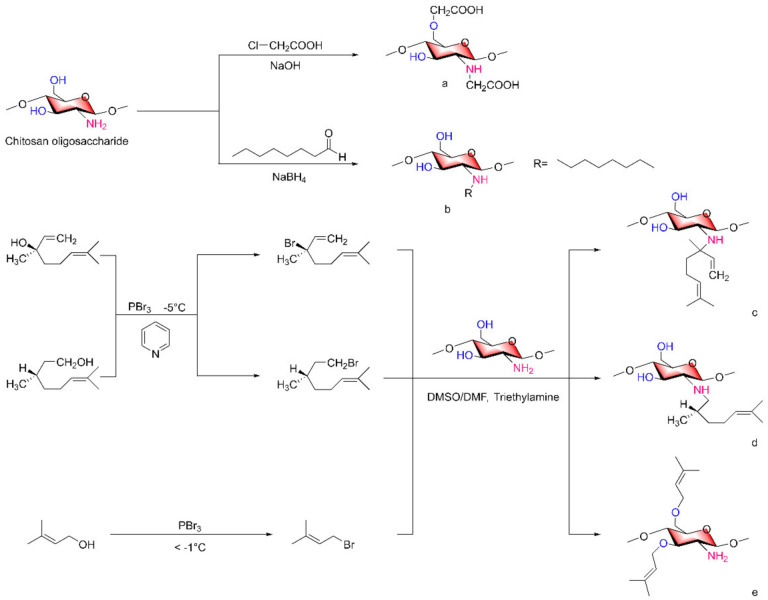
Synthesis of alkylated chitosan oligosaccharide derivatives. (**a**) carboxymethyl COSs, (**b**) octane COSs, (**c**) alkylated COS derivatives Lin-g-COS, (**d**) alkylated COS derivatives Cit-g-COS, (**e**) COS-O-Ter derivatives.

Meirong Huo et al. [37] reported methods for synthesizing carboxymethyl COSs (Figure 3a) or octane COSs (Figure 3b). COSs react with chloroacetic acid in an alcohol solution of NaOH to form carboxymethyl structures on -NH_2_ and 6-OH groups to form carboxymethyl COSs. N-octane COSs were formed by the reaction of COSs and octyl aldehyde to form Schiff base intermediates in the presence of sodium borohydride, a strong reducing agent.

When the alkylation reagents contain hydroxyl groups, the alcohols can first be converted to halogen substituents and then reacted with COSs to obtain the corresponding alkylated chitosan derivatives.

In a study by Shuifang Mao et al. [38,39], linalanol (Lin) and citronellol (Cit) were reacted with PhBr_3_ at −5 °C under a pyridine-catalyzed reaction for a certain time to form their respective brominated compounds Lin-Br and Cit-Br, which were then reacted with COSs under triethylamine as a catalyst, to obtain the alkylated COS derivatives Lin-g-COS (Figure 3c) and Cit-g-COS (Figure 3d) on the amino group at 50 °C. Similarly, Lin Yue et al. [40] reacted terpenol (Ter) with bromine and then with the hydroxyl group of COSs to form COS-O-Ter derivatives (Figure 3e). It was noted in this literature that benzaldehyde can be used to protect the amino group of COSs before the reaction of COSs with bromine compounds.

### 2.4. Quaternized COS Derivatives

According to the existing literature, the chemical structure of polysaccharides, including chitosan, after quaternary ammonium modification shows an increase in positive charge, and chitosan already contains a positive amino charge. Due to the increase in cationic charge density, the interaction between polysaccharides and substances with a negative charge surface increases [41]. Based on this conclusion, the charge density of COSs can be further improved by the quaternization of COSs. Quaternized COS derivatives can not only form quaternary structures directly on the amino group but also introduce substances with quaternary structures.

**Figure 4:**
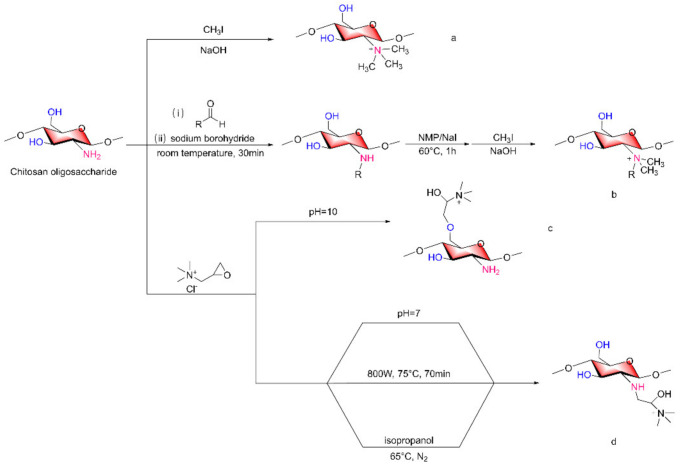
Synthesis of quaternized chitosan oligosaccharide derivatives. (**a**) N, N, N-quaternized COSs, (**b**) more diversified N-quaternized COSs, (**c**) pH = 10, glycidyl trimethylammonium chloride can introduce quaternary ammonium groups in 6-OH of COSs, (**d**) pH = 7 or other conditions, glycidyl trimethylammonium chloride can introduce quaternary ammonium groups in -NH_2_ of COSs.

The quaternization of COSs can be achieved on both amino and hydroxyl groups. In general, CH_3_I/NaOH treatment of COSs can yield amino quaternization, such as in N, N, N-trimethyl COSs (TMCs, Figure 4a), and the quaternization group prepared by this method is characterized by three methyl groups attached to the N atom. If substituent quaternization inconsistencies are desired on the N atom, reductive alkylation followed by methylation can be implemented [42]; substituted alkyl groups on N atoms depend on the alkyl structure of the aldehydes selected for reductive alkylation, so more diversified N-quaternized COSs can be obtained (Figure 4b). In addition, according to the pH of the reaction conditions, epoxides such as glycidyl trimethylammonium chloride can introduce quaternary ammonium groups in different positions when reacting with COSs (Figure 4c,d) [43,44].

Bo Liu et al. [45] used microwave-assisted synthesis of quaternary ammonium carboxymethyl COSs (Figure 4d), which was carried out at 800 W and 70 °C for 25 min using chloroacetic acid as a modifier, a 2,3-epoxypropyltrimethyl ammonium chloride quaternization reaction was then carried out at 800 W and 75 °C for 70 min prior to product purification. The carboxymethyl substitution degree was 0.88, and the quaternary ammonium salt substitution degree was 0.75. Feng-Xiang Tang et al. [46] reacted COSs with 1-[4-(2-carboxyethyl) phenoxy] zinc (II) phthalocyanine, during which the COSs were added to isopropanol under N_2_ and heated to 65 °C (Figure 4d). Then, a mixture of 2,3-epoxypropyl ammonium chloride was dissolved in isopropanol, the reaction was allowed to proceed for 8 h, and the quaternization reaction took place on the amino group that had not been replaced.

### 2.5. Other COS Derivatives

**Figure 5:**
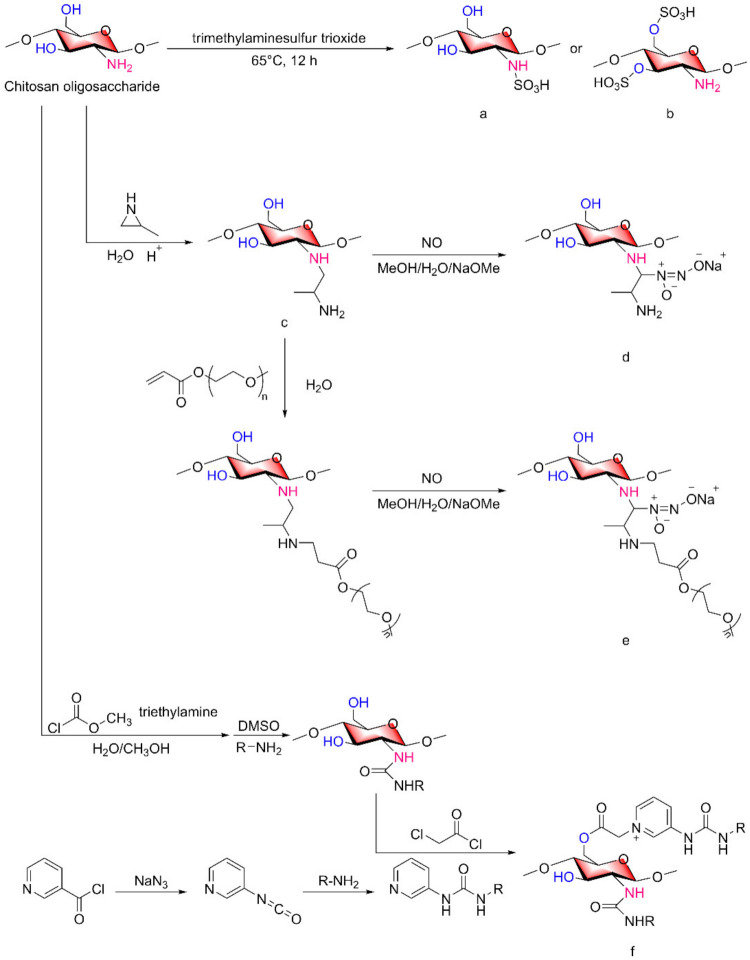
Synthesis of other varieties of chitosan oligosaccharide derivatives. (**a**) N-sulfated COSs, (**b**) 3-O-sulfated COSs, (**c**) grafting of 2-methyl aziridine onto primary amines of COSs, (**d**) N-diazeniumdiolate formation of COSs, (**e**) PEGylation of 2-methyl aziridine-grafted-COS with NO donor functionalization, (**f**) 2,6-diurea-COS derivative.

A variety of COS derivatives have been extensively studied. However, some COS derivatives have not been clearly studied even though they have certain biological activities. The functional characteristics of sulfated polysaccharides are related to the distribution and quantity of sulfated groups on the molecular chain, and sulfated COSs have many biological effects. The copolymer of COSs, anhydrous sodium carbonate and trimethylammonium sulfur trioxide reacted at 65 °C for 12 h and then was dialyzed and lyophilized to obtain sulfated COSs (Figure 5a,b) [47,48].

Amino diazo derivatives of COSs, similar to quaternized COSs, can be classified into two groups: one group is the formation of diazo structures directly on the amino group, and the other group is the introduction of compounds with diazo structures.

Yuan Lu et al. [49]. reported a method for the preparation of COS derivatives that can release NO. After grafting of 2-methyl aziridine onto primary amines of chitosan oligosaccharides (Figure 5c), two schemes were adopted, one is to directly generate N-diazeniumdiolate (Figure 5d), the other is PEGylation of 2-methyl aziridine-grafted-chitosan oligosaccharide with NO donor functionalization (Figure 5e). Similarly, Katelyn P. Reighard et al. [50] used a similar method to synthesize chitosan oligosaccharide derivatives capable of releasing NO. After 2-methyl aziridine was grafted onto primary amine, by the Michael addition of acrylates to the newly produced primary amino to form structurally distinct saturated ester functional groups on the scaffold backbone.

Monosubstituted COS derivatives, which only undergo change on an amino group or a hydroxyl group, are not currently popular, and the active groups on the sugar chain cannot be fully utilized, which leads to a lack of application. Multiple modified COSs show a trend of development in the current research. When a certain group is introduced into the main chain, the remaining amino or hydroxyl groups that have not been replaced can continue to combine with other target substances to achieve the efficient utilization of multiple benefits of a substance.

Jingjing Zhang et al. [51] prepared 2-urea-COS derivatives and 2,6-diurea-COS derivatives (Figure 5f) by 2-methoxyformylated of COSs. By changing the material represented by the R group, eight of the above substances were obtained.

## 3. Derivation on a Protective Basis

The reactions mentioned above, such as acetylation and quaternization, as nonselective reactions, cannot be controlled in terms of which group participates in the reaction. Under certain reaction conditions, two active groups will inevitably react at the same time. To obtain selective COS derivatives, it is necessary to shield one of the active groups of COSs with a protective group before the synthesis of the final product and then release the protective group when the final product is obtained.

**Figure 6:**
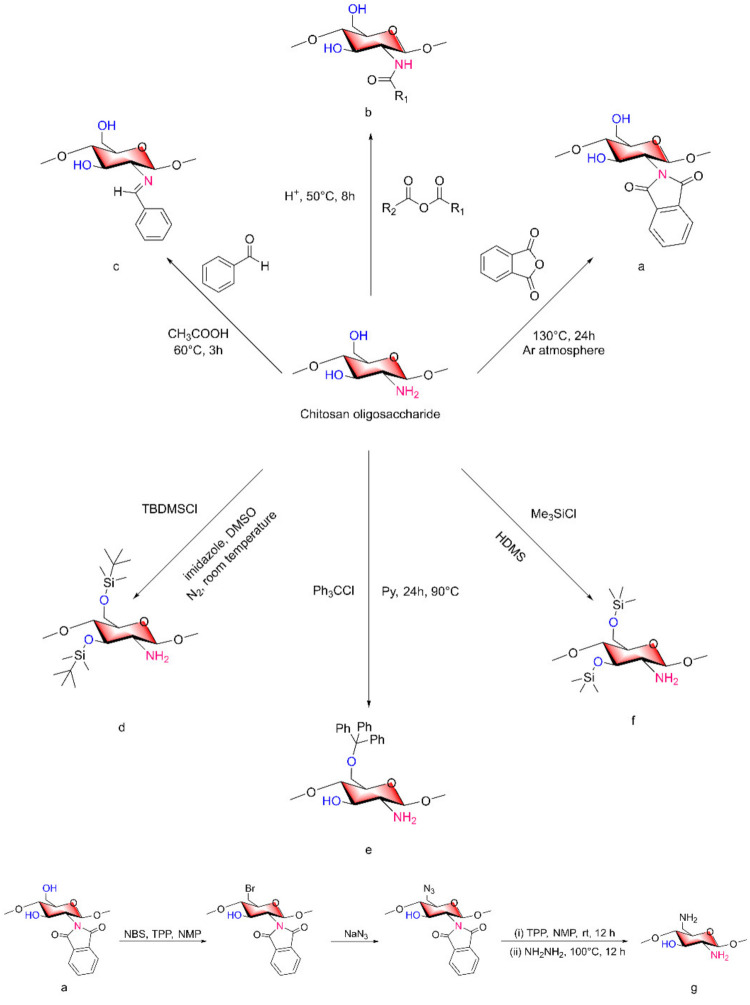
Synthesis of common protective groups of COSs. (**a**) the amino groups of COSs were protected by phthalic anhydride to form an imide structure, (**b**) N-acylated COSs, (**c**) N-benzylidene of COSs, (**d**) 3,6-O-di-tertiarybutyldimethyls-COSs, (**e**) 6-O-triphenylmethyl COSs, (**f**) 3,6-O-di-trimethylsily COSs, (**g**) 2,6-diamino-chitosan.

In organic synthesis, acylation reactions are often used for the protection of amino groups, and the form of acylation is usually that resulting in an acetamide or imide (Figure 6a,b). In a paper on the synthesis of chitosan derivatives published by Zhangyong Si [52], 2,6-diamino-chitosan (2,6-DAC) was synthesized. First, low-MW chitosan and anhydrous DMF were subjected to bath sonication at 80 °C and then reacted with phthalic anhydride at 130 °C for 24 h. NMR analysis revealed that more than 96% of the amino groups were protected by phthalic anhydride to form an imide structure (Figure 6a). Phthalic anhydride acted as the protective group of the amino groups in this step. After amino group protection, N-phthaloyl chitosan was mixed with N-bromosuccinimide (NBS), triphenylphosphine (TPP) and N-methyl-2-pyrrolidone (NMP) to obtain 6-bromo-N-phthaloyl chitosan. After the reaction with NaN_3_, the bromine at position 6 in the chitosan was azidated. After reacting with TPP and NMP for 12 h at room temperature, hydrazine was used for 12 h at 100 °C to remove the protective group and obtain 2,6-DAC as the final product [52] (Figure 6g).

When it is necessary to retain the hydroxyl structure and modify only the amino group, tert-butyldimethylsilyl (TBDMS) chloride, triphenylmethyl chloride and trimethylsilyl chloride can be used to protect the hydroxyl group in COSs (Figure 6d–f) [53]. Triphenylmethyl chloride is a special reagent because it can only form a triphenyl methyl structure with 6-OH on COSs, while the other two reagents can form protective groups for 3-OH and 6-OH. Priyanka Sahariah and Vivek S. Gaware [54] also formed silane ether to protect the hydroxyl group, reacted it to form a variety of chitosan derivatives with the structure of quaternary ammonium salt, and then removed TBDMS. Berglind E. Benediktsdóttir used [55] di-TBDMS chitosan as a precursor for the synthesis of TMC homopolymer and highly substituted N-alkyl-N,N-dimethyl chitosan derivatives to obtain the required product and then remove the protective group. Priyanka Sahariah [56] synthesized three guanidinylated chitosan derivatives using TBDMS protective groups. The TBDMS on the prepared guanidinylated chitosan was removed, and the hydroxyl group was free again. Finally, chitosan derivatives with only amino group changes were obtained.

When the TBDMS group is used as the protective group, hydrochloric acid/alcohol (methanol, ethanol) is generally used as the final removal method at room temperature. The method of removing a protective group in acidic environments may cause the cleavage of glycosidic bonds, and the removal efficiency is not high. Therefore, TBAF/NMP can be used instead of removing the protective group, and it is more efficient [55,57].

In addition, Lin Yue et al. [40] synthesized the corresponding N-benzylidene of COSs according to the previous Schiff base method under some modifications (Figure 6c) to protect the -NH_2_ groups of COSs.

The protective groups of the above chitosan derivatives can be removed to restore the original structure of chitosan.

## 4. Antibacterial Characteristics of COSs

### 4.1. Mode of Action of COSs as Antibacterial Agents

The cell wall of gram-positive bacteria consists of negatively charged teichoic acid (TAS) and peptidoglycans that are tens of nanometers thick [58,59]. Teichoic acid is a linear polyanionic phosphate-rich polymer composed of wall teichoic acid (WTA) and lipoteichoic acid (LTA). WTA is covalently linked to peptidoglycan, the glycolipid of LTA is fixed on the membrane, and its glycerol phosphate chain extends to the membrane wall [60]. A matrix formed of peptidoglycan and teichoic acid interacts with divalent metal cations to maintain homeostasis. According to the structure of LTA and its synthesis in vivo, LTA can be used as an anti-infection target [61]. On the surface of gram-negative bacteria, similar to gram-positive bacteria, peptidoglycan is present covered with an outer membrane composed of lipoprotein and lipopolysaccharides (LPSs). LPS molecules are rich in negatively charged lipid A, which is also stabilized by divalent cations (e.g., Mg^2+^ and Ca^2+^) [62].

**Figure 7:**
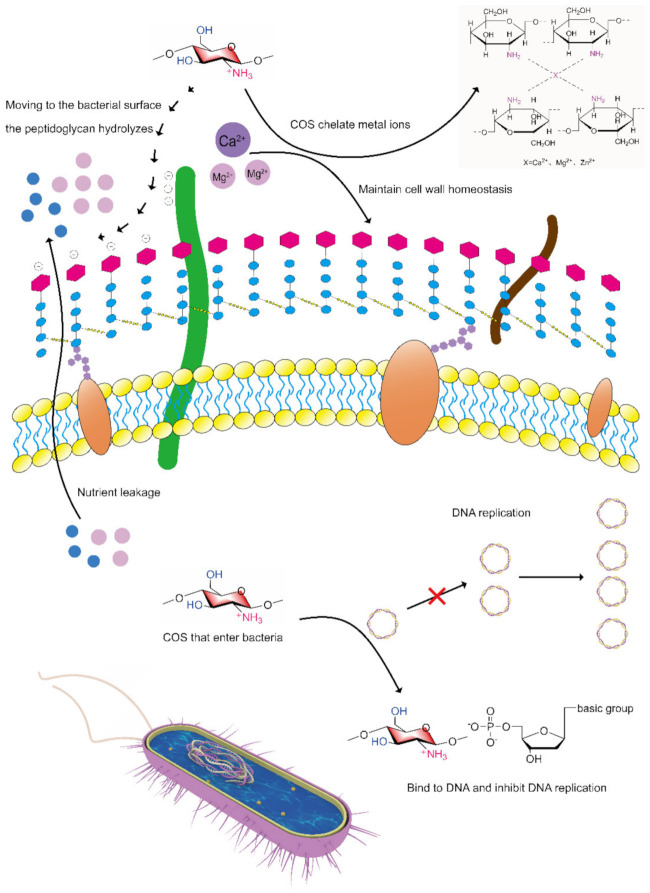
Mechanism of COSs against gram-positive bacteria.

The following mode of action of chitosan or COSs as antibacterial agent have been proposed. First, it is generally believed that the protonated amino groups on COSs bind to negatively charged groups on the surface of microorganisms, which is the key mode of action of their antibacterial activity [53]. COSs with positive amino charges will move to the surface of negatively charged bacteria due to electrostatic action, resulting in the hydrolysis of peptidoglycan indicated by gram-positive bacteria and the leakage of the intracellular contents. The second proposed mode of action, involving the outer membrane permeability of gram-negative bacteria, suggests that COSs can influence the transport of nutrients to cells by gram-negative bacteria in the form of ionic bonds. In the third mechanism, COSs cross cell membranes and cytoplasm and interact with bacterial DNA, preventing it from being transcribed. Finally, a proposed mode of action is that where metal ions present in the bacterial surface are chelated by the amino groups of chitosan. This chelation effect is found to overpower the electrostatic effect when the pH of the medium is higher than the pKa of chitosan [63]. When bacteria undergo amino chelation, the stability of bacterial cell walls is destroyed, exhibiting antibacterial effects. Figure 7 shows the mechanism of COSs against gram-positive bacteria.

In the abovementioned antibacterial mode of action, both the protonation of amino groups and the formation of the metal complexes depend on unshared electron pairs on amino groups. The formation of amino derivatives of COSs can change the electron cloud density on the amino group, or by introducing other antibacterial molecules into active functional groups to form COS derivatives with dual antibacterial sites.

### 4.2. Characteristics Affecting Antibacterial Activity

Some studies have been reported that the antibacterial activity of COS derivatives is affected by the modification site, the chemical properties of the introducing group and the degree of substitution on the main chain [53]. When the cationic charge density at the modification site changes, the binding of COSs with the bacteria surface negative charge will be more obvious, and the interaction binding will also be stronger [64].

Based on the chemical modification of COSs, the antibacterial activity of COSs can be improved [65,66,67]. In addition to the change in cationic charge, some studies have been reported that antibacterial molecules can be introduced into the main chain of COSs to enhance the antibacterial efficacy [68].

**Figure 8:**
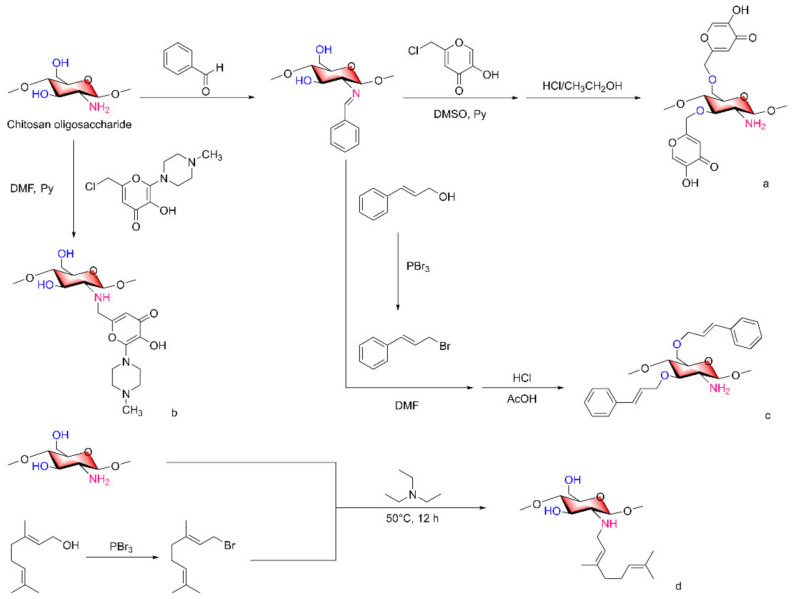
Antibacterial molecules combined with COSs. (**a**) COS/kojic acid graft complexes, (**b**) COS-N-chlorokojic acid mannich base, (**c**) COS-O-Cin, (**d**) COS-Ger derivative.

Some research teams have published relevant papers to verify that COSs can improve their antibacterial activity by reacting them with antimicrobial molecules to form derivatives [69]. Kojic acid has antibacterial and antiviral effects [70], and kojic acid was grafted onto the 3,6-OH of COSs to form COS/kojic acid graft complexes (Figure 8a) [71]. Through antibacterial experiments, the authors indicated that the antibacterial activity of COS/kojic acid was improved due to the introduction of a 5-hydroxypyranone group on the main chain. In addition, a nontoxic antimicrobial material based on a COS-N-chlorokojic acid mannich base (COS-N-MB, Figure 8b) was synthesized by a selective partial alkylation reaction [24]. Cinnamyl alcohol (Cin) can be used in the synthesis of antimicrobial agents, and the reaction of Cin with the hydroxyl group on COS C6 to form COS-O-Cin (Figure 8c) was compared with the solution of COSs, Cin and sodium benzoate at the same concentration [26]. The team chose to react the COS hydroxyl groups to form derivatives to retain the amino activity of COSs while improving their antimicrobial activity. Geraniol (Ger) is an acyclic monoterpene compound that shows antibacterial activity [72]. Ran Bi et al. [73], overcame the shortcoming of coupling antibacterial molecules to 3,6-hydroxyl groups, synthesized a novel COS derivative with Ger moiety through a simple method, and evaluated their antibacterial activity (Figure 8d).

In the COS-Ger derivative, the oxygen substitution showed a stronger antibacterial effect than amino substitution. The results of antibacterial experiments showed that the antibacterial activities of the above derivatives against *Escherichia coli* and *Staphylococcus aureus* were significantly higher than that of COSs, the antibacterial activities increased with increasing substitution degree, and the antibacterial activity against *S. aureus* was more obvious than that against *E. coli*. The difference in the antibacterial activity between the two types of bacteria may be due to the structural differences in the cell walls of gram-positive and gram-negative bacteria.

When antibacterial molecules combined with COSs, unsubstituted amino group and antibacterial molecules work together. This can also explain why the same antibacterial molecule attached to the 3,6-hydroxyl group of COSs is more effective than when attached to the amino group because the hydroxyl group can be replaced with more free amino groups, which can also play an antibacterial role and is consistent with the results of the above study [73].

In particular, similar to the molecular structure of COS-N-MB, the derivative exhibits a variety of antibacterial mode of actions in that the unsubstituted amino group is protonated and attracted to the bacterial surface by a negatively charged group due to electrostatic action. The active amino group and 5-hydroxypyranone complex with Ca^2+^ and Mg^2+^, damaging the cell wall. In addition, COS-N-MB can also cross the peptidoglycan layer and bind with DNA to inhibit protein synthesis and gene expression in bacteria, eventually leading to bacterial death. In conclusion, based on the molecular structure of COSs and according to the characteristics of the newly introduced antibacterial molecules, the formation of new derivatives can not only exhibit better antibacterial activity than the original derivatives but also show synergistic effects. A variety of antibacterial mode of actions work together, which has great potential to overcome bacterial resistance.

## 5. Antitumor Activity of COSs

At present, the research related to the antitumor characteristics of COSs mainly focuses on the chemical modification of COSs to improve their antitumor activity and the use of COS derivatives as drug carriers for the targeted transport of antitumor drugs.

### 5.1. Antitumor Activity

The antitumor activity of COSs is related to their physical and chemical properties, including Molecular Weight (MW), Degree of polymerization (DP), Deacetylation Degree (DD), charge distribution and chemical modification. To investigate the antitumor activity of COSs, the influence of MW should also be taken into consideration in addition to the functional groups on the pyranose ring, including amino and acetyl amino groups [74]. Some studies have evaluated the antitumor differences between dimeric and hexameric COSs, with hexameric COSs showed the best effect on A549 cells [75]. In vitro studies have shown that COSs could exhibit antitumor activity on a variety of tumor cells, and in vivo experiments have shown that COSs could inhibit tumor proliferation and metastasis [76].

Ronghua Huang et al. [77] believed that COS derivatives with a high charge could significantly reduce the survival ability of tumor cells, and suggested that the induction of tumor cell necrosis was the main antitumor mechanism. That is, shell oligosaccharide antitumor activity is closely related to the intensity of the positive charge. When the DD of COSs increases on the sugar chain, the positive charge will increase correspondingly, increasing the interactions with tumor cells; in other words, when the positive charge density on the pyranose ring is changed, the antitumor activity of the COSs will be improved. COSs with a reactive group can support this strategy very well.

In 2020, Zhiwen Jiang et al. [78] found that carboxymethyl COSs exhibited antitumor activity on BEL-7402 cells in vitro and H22 sarcoma cells in vivo through multiple mechanisms including inhibition of proliferation, elevation of the immunity index, induction of cell apoptosis and necrosis, while these compounds had no toxic effects on L-02 cells in normal liver and other organs.

**Figure 9:**
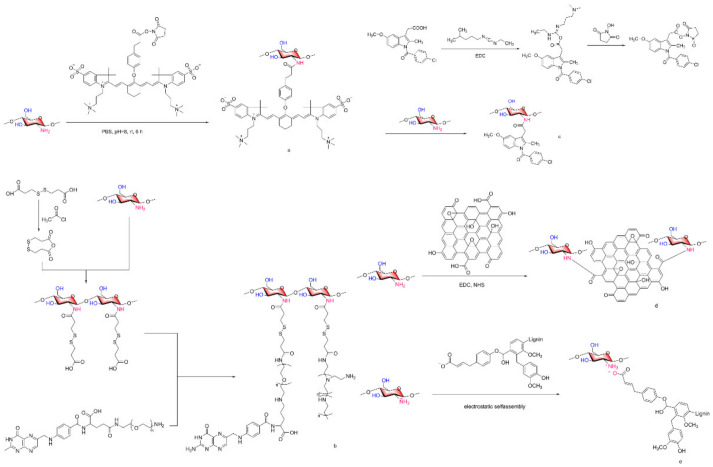
Synthesis of chitosan oligosaccharide nanoparticles for tumor-targeted drug delivery. (**a**) COL-ZW800-1 conjugate, (**b**) FA-PEG-CS-PEI, (**c**) COS-IDM, (**d**) nGO-COS, (**e**) enzymatic hydrolysis lignin (EHL)/COS.

It has been reported that COS lactate (COL) has great potential for tumor imaging due to its highly efficient intracellular and site-specific delivery [79,80]. Photothermal therapy (PTT) has great potential in tumor therapy because of its low toxicity to normal tissues and high tumor ablation efficiency [81,82,83]. Sungsu Lee et al. [84] reported a study on the tumor targeting property of COL and the advantages of PTT, combining COL with near-infrared (NIR) fluorescent group ZW800-1 to form a COL-ZW800-1 (Figure 9a) conjugate for targeted cancer imaging and PTT in cancer treatment. The positive charge on COL binds the COL-ZW800-1 conjugate to the outer surface of the negatively charged tumor cell membrane. The COL-ZW800-1 conjugate showed strong absorbance in the NIR region, with tumor specific targeting, high photothermal conversion efficiency and an effective tumor ablation effect.

### 5.2. Tumor-Targeted Drug Systems

To date, chemotherapy is still the main choice for the treatment of tumors, but due to the existence of multiple drug resistance (MDR), the therapeutic effect has been unsatisfactory. There are two types of MDR, intrinsic resistance and acquired resistance. One of the most common mechanisms of acquired drug resistance relies on drug efflux of cancer cells mediated by ATP binding box (ABC) transporters [85]. P-Glycoprotein (P-gp) is one of the major transporters that expels many drugs, reducing intracellular drug concentrations below effective cytotoxic thresholds [86,87,88].

Polymer nanoparticles can be used to deliver drugs and/or genes to tumor sites [89]. The high viscosity and poor solubility of chitosan at physiological pH (7.2–7.4) are the main obstacles to its successful application in gene vectors. COSs have attracted great interest of researcher because of their good water solubility and biocompatibility, but their applications in gene transfer are limited due to their low cationic charge density and proton sponge effect. COSs have active hydroxyl groups and amino groups, and further cation modification can effectively improve their gene transfer ability [90,91].

Therefore, in the relevant studies on the use of COSs in the treatment of tumors, COSs and their derivatives are used as anticancer drugs, additionally as drug carriers besides for direct modification to achieve the targeting of tumors and improve the efficacy of anticancer drugs, which is also a major research hotspot in the application of COSs in tumor treatment.

Lejiao Jia et al. [92] constructed novel folic acid-grafted COS disulfide-containing polyethyleneimine copolymer-based silica nanohybrids (FA-PEG-CS-PEI/SN, Figure 9b) for the simultaneous delivery of p-shRNA and paclitaxel (PTX), and plasmid DNA expressing shRNA was used to interfere with p-glycoprotein. PTX was dispersed into pH-responsive CTAB micelles to form PTX-loaded silica nanoparticles. The results showed that the target mRNA showed high gene silencing efficiency, downregulated P-gp expression, and increased intracellular drug concentration. PTX and p-shRNA composite nanoparticles significantly inhibited the proliferation of MCF-7/ADR cells, and the resistance to PTX was completely reversed. Jae-Young Lee et al. [93] also constructed COS nanoparticles for the targeted treatment of tumors. Indomethacin was activated by EDC and reacted with NHS to generate IDM-NHS, which was then linked with the amino group of COSs through an amide bond to form COS-IDM (Figure 9c). Due to the amphiphilic properties of COS-IDM, hydrophobic doxorubicin (DOX) was loaded into the inner core of the nanocarrier by self-assembly. The “chemical sensitization effect” of indomethacin enhances the toxicity of DOX to tumor cells, and the formation of COS nanoparticles ensures the effective targeting of low water-soluble anticancer drugs. Compared with free IDM administration, IDM coupled to COS can prolong the blood circulation of DOX, thus improving the tumor targeting efficiency of DOX [93]. To improve the biocompatibility of nanoscale graphene oxide (nGO) and the targeting of dacarbazine (DITC), Xiaozhen Zhan et al. [94] constructed COSs and anti-CD46 antibody nanoparticles jointly constructed by nGO and loaded with DITC, to form nGO-COS-CD47/DTIC nanocomposites (Figure 9d). The nanocomposite exhibits controlled release behavior of pH dependence and laser irradiation dependence, and has remarkable tumor targeting specificity, stable self-fluorescence properties and excellent photothermal efficiency.

The microenvironment in which a tumor is located has many different characteristics than that in normal physiological tissue [95]. Tumor microenvironment-based pH-based nanoparticles are one of the most studied stimulus-responsive drug nanocarriers and are sensitive to acidic environments inside and outside tumor cells [96,97,98]. Some modified COSs as carriers have been widely studied, operating according to the changes in the microenvironment to form a pH-specific response to release antitumor drugs.

Kai Chen et al. [99] formed enzymatic hydrolysis lignin (EHL)/COS nanoparticles (Figure 9e) by electrostatic self-assembly between the amino group of COSs and the carboxyl group of EHL and loaded cytarabine (Ara-C) into them. The nanoparticle-stabilized emulsions contained curcumin and stably loaded drugs of different polarities into high internal-phase Pickering emulsions (HIPPEs), and the adverse reactions caused by drug-drug interactions (DDIs) were reduced. This system showed good pH responsiveness release in the tumor environment. In vitro release experiments showed that the activity of Ara-C and curcumin combined with HIPPEs against Jurkat cells was twice that of Ara-C or curcumin alone with HIPPEs, suggesting synergistic therapeutic effects.

Xia Chen et al. [95] reported a preparation method of dual prodrug nanoparticles based on COSs for enhanced tumor targeted drug delivery. Nanoparticles can effectively target CD44 receptors on the surface of tumor cells, and DOX and oleanolic acid (OA) can be released from the nanoparticles in a sustainable and pH-responsive manner, thus enabling specific drug release triggers in the tumor microenvironment. The results of fluorescence microscopy showed that the antitumor activity of the nanoparticles combined with the two prodrugs was better than that of the free drugs.

**Figure 10:**
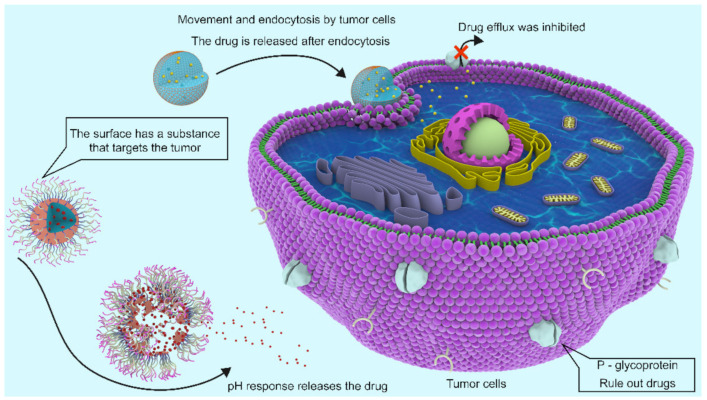
Two kinds of COS nanocapsules act on tumor cells.

Compared with free drugs, anticancer drugs loaded in COS carriers can realize the targeted delivery of antitumor drugs and release drugs in response to a specific tumor microenvironment, thus improving the bioavailability of antitumor drugs and effectively overcoming the insufficient therapeutic effect and serious side effects caused by MDR. Similarly, when COSs were used as drug carriers, the modified COS nanoparticles showed higher drug delivery efficiency and more specific anticancer drugs targeting cancer cells than the unmodified COS nanoparticles [100]. Figure 10 shows the two kinds of COS nanocapsules act on tumor cells.

## 6. Future Perspectives

Chitosan oligosaccharides obtained from marine sources are abundant, exhibit a variety of biological activities and modifiability, have great development potential and will still be studied in the future. In the future, shell-derived COSs with a MW less than 1000 Da should be studied, especially those with different DPs. Components with the best biological activity should be determined, and the effect of the DP should be studied. The impacts of these factors are still unknown in the study of COSs. In addition, laboratory- or industrial-scale production of COSs using the most degradation methods cannot easily yield a large amount of product with a single DP. If glucosamine is used as a reactant, the catalyst can be directed to form β-1,4 glycosidic bonds and can form a specific DP. In this way, the problem of unstable MW distribution in the production process may be solved. Judging from the current research on carbohydrate synthesis, there is hope of success in COS development in future research.

## 7. Conclusions

In this paper, the synthesis methods of common derivatives of COSs and some derivatives with little research but development potential are reviewed, and the protective groups selected for the selective reaction of COSs are described. The use of protective groups can improve the utilization efficiency of COSs. The antitumor application of COS derivatives and some new research results are described. The active groups of COSs provide the possibility for the synthesis of a variety of derivatives, and their biological activities are closely related to their physical and chemical properties. The amino group with a cationic charge on COSs can not only exhibit antibacterial action alone but also play a synergistic role with the antimicrobial molecules introduced on the sugar chain. In terms of antitumor activity, COS derivatives have antitumor activity, and the construction of COS carrier-loaded drugs can improve the drug efficacy and overcome the disadvantages of current antitumor drugs. With further research, the understanding of COSs will be improved in the future.
